# Threats to Global Mental Health From Unregulated Digital Phenotyping and Neuromarketing: Recommendations for COVID-19 Era and Beyond

**DOI:** 10.3389/fpsyt.2021.713987

**Published:** 2021-09-14

**Authors:** Hossein Akbarialiabad, Bahar Bastani, Mohammad Hossein Taghrir, Shahram Paydar, Nasrollah Ghahramani, Manasi Kumar

**Affiliations:** ^1^Research Center for Psychiatry and Behavioral Sciences, Department of Psychiatry, School of Medicine, Shiraz University of Medical Sciences, Shiraz, Iran; ^2^Student Research Committee, Shiraz School of Medicine, Shiraz University of Medical Sciences, Shiraz, Iran; ^3^Medicine-Nephrology, Saint Louis University School of Medicine, Saint Louis, MO, United States; ^4^Trauma Research Center, Shahid Rajaee (Emtiaz) Trauma Hospital, Shiraz University of Medical Sciences, Shiraz, Iran; ^5^Division of Nephrology, Department of Medicine, Penn State University College of Medicine, Hershey, PA, United States; ^6^Department of Psychiatry, University of Nairobi, Nairobi, Kenya; ^7^Department of Clinical, Educational and Health Psychology, University College London, London, United Kingdom

**Keywords:** digital phenotyping, digital neuromarketing, global mental health, data privacy, digital mental health regulations, lower and middle income counteries

## Abstract

The new era of digitalized knowledge and information technology (IT) has improved efficiency in all medical fields, and digital health solutions are becoming the norm. There has also been an upsurge in utilizing digital solutions during the COVID-19 pandemic to address the unmet mental healthcare needs, especially for those unable to afford in-person office-based therapy sessions or those living in remote rural areas with limited access to mental healthcare providers. Despite these benefits, there are significant concerns regarding the widespread use of such technologies in the healthcare system. A few of those concerns are a potential breach in the patients' privacy, confidentiality, and the agency of patients being at risk of getting used for marketing or data harnessing purposes. Digital phenotyping aims to detect and categorize an individual's behavior, activities, interests, and psychological features to properly customize future communications or mental care for that individual. Neuromarketing seeks to investigate an individual's neuronal response(s) (cortical and subcortical autonomic) characteristics and uses this data to direct the person into purchasing merchandise of interest, or shaping individual's opinion in consumer, social or political decision making, etc. This commentary's primary concern is the intersection of these two concepts that would be an inevitable threat, more so, in the post-COVID era when disparities would be exaggerated globally. We also addressed the potential “dark web” applications in this intersection, worsening the crisis. We intend to raise attention toward this new threat, as the impacts might be more damming in low-income settings or/with vulnerable populations. Legal, health ethics, and government regulatory processes looking at broader impacts of digital marketing need to be in place.

## Threats to Global Mental Health from Unregulated Digital Phenotyping and Neuromarketing: Recommendations for COVID-19 Era and Beyond

Globally stigma, gender-, cultural-, racial insensitivities, and discrimination are some of the systemic barriers in optimally scaling up mental health care (1). Digital tools have the potential to ease these barriers by making mental health services accessible to all; more importantly to remote, needier, and vulnerable populations. Digital solutions offer more choices to patients and service users. Amidst the COVID-19 pandemic, digital health and telehealth services have expanded tremendously to meet the rising demands (2–5). Using such technologies has improved freedom, efficacy, and flexibility in communication for patients and physicians worldwide. On the flip side, several studies have shown that digital mental health applications may alienate some and raise anxiety and stress for some clients, including fear of relapse and even paranoid thinking (6–9). Concerns that continue to remain unaddressed include breach of patients' privacy, confidentiality, autonomy, and undermining of patients' agency by using patient data for other purposes than they consented for (10). The rapid albeit heedless use of digital tools has introduced a substantial threat. There is a relative lack of proper legislation and practical safeguards to protect patients' personal information in general and more so in emerging economies (11). We underscore the emergence of a potentially serious threat to individuals and communities at large arising from the intersecting impact of rapid “digital phenotyping” and “digital neuromarketing” globally. This commentary collectively highlights how this unregulated practice may adversely impact under-resourced contexts where patient interests may be cast aside to serve big data for commercial gains.

Digital phenotyping aims to detect individual behavior, activities, interests, and physiological features to utilize the information to customize the care (12). A few examples are: tracking the digital biomarkers like locations, accelerometer, social communication, screen lock/unlock events, call logs, camera events, use of particular app(s), browser history, light sensor, sleep-wake cycle, exercise, and social interactions through the smartwatches/phones (13). Digital phenotyping applications have been utilized in detecting and intervening in a wide range of psychiatric disorders, from mood and anxiety disorders to drug abuse and suicidal thoughts (14–21). Recently, sensing technologies have yielded a wide range of usage in detecting and predicting the psychological and psychiatric conditions. Using heart rate variability (HRV) by multilayer perception model, skin conductance (SC) method, and long short-term memory neural network models (LSTM) are the commonest ways currently used for detecting and predicting the stress level through biosensors (22). Apart from sensors, other minor aspects of smartphones such as the average number of daily calls and text messages, average time spending on social media and entertainment applications, and the average time of web browsing have been shown to be able to detect and predict the severity of depression with high accuracy (23). However, alongside the competitive advantage of offering appropriate customized services, digital phenotyping intrinsically impacts what it is to be a human person and potentially undermines human-human interaction as emanating from a therapeutic/clinical consultation where two individuals (at times more) connect deeply to address highly intricate and complex problems (24–27).

Neuromarketing, too, has similarly attracted significant academic and commercial interest (28). It develops tools to capture the unspoken feelings/emotions, desires, and cognition of the consumers to various marketing stimuli to foresee personal decisions, such as purchasing decisions. The neuromarketing studies took advantage of the advances in the neurosciences, and while in the beginning, they were more focused on neuroimaging, encompassing functional brain MRI (fMRI) and electroencephalography (EEG), lately the field has turned to tapping into autonomic nervous system to enhance targeted marketing using biofeedback mechanisms (29). For example, using digital neuromarketing, Cerf and colleagues predicted the future sale of a movie by measuring the engagement of a sample population while simultaneously looking to their EEGs by around 20% better comparing to the traditional methods (30, 31); Moreover, there are multiple other real world implications of neuromarketing are available (32, 33). Furthermore, neuromarketing research has recently turned to detecting clients' physiologic responses, such as heart rate changes, eye tracking, galvanic cutaneous reactions, and developing facial action coding systems, with or without utilizing brain signal recording techniques gauging occult clients' reactions (30). Digital neuromarketing is quite different from traditional marketing tools since it bypasses the clients' thinking processes and directly captures the response of the clients' nervous systems (34). The market of global neuromarketing is by no mean negligible; the market was valued 1.158 billion US dollar in 2020 and projected to be 1.896 billion dollar in 2026 (35).

**Figure 1:**
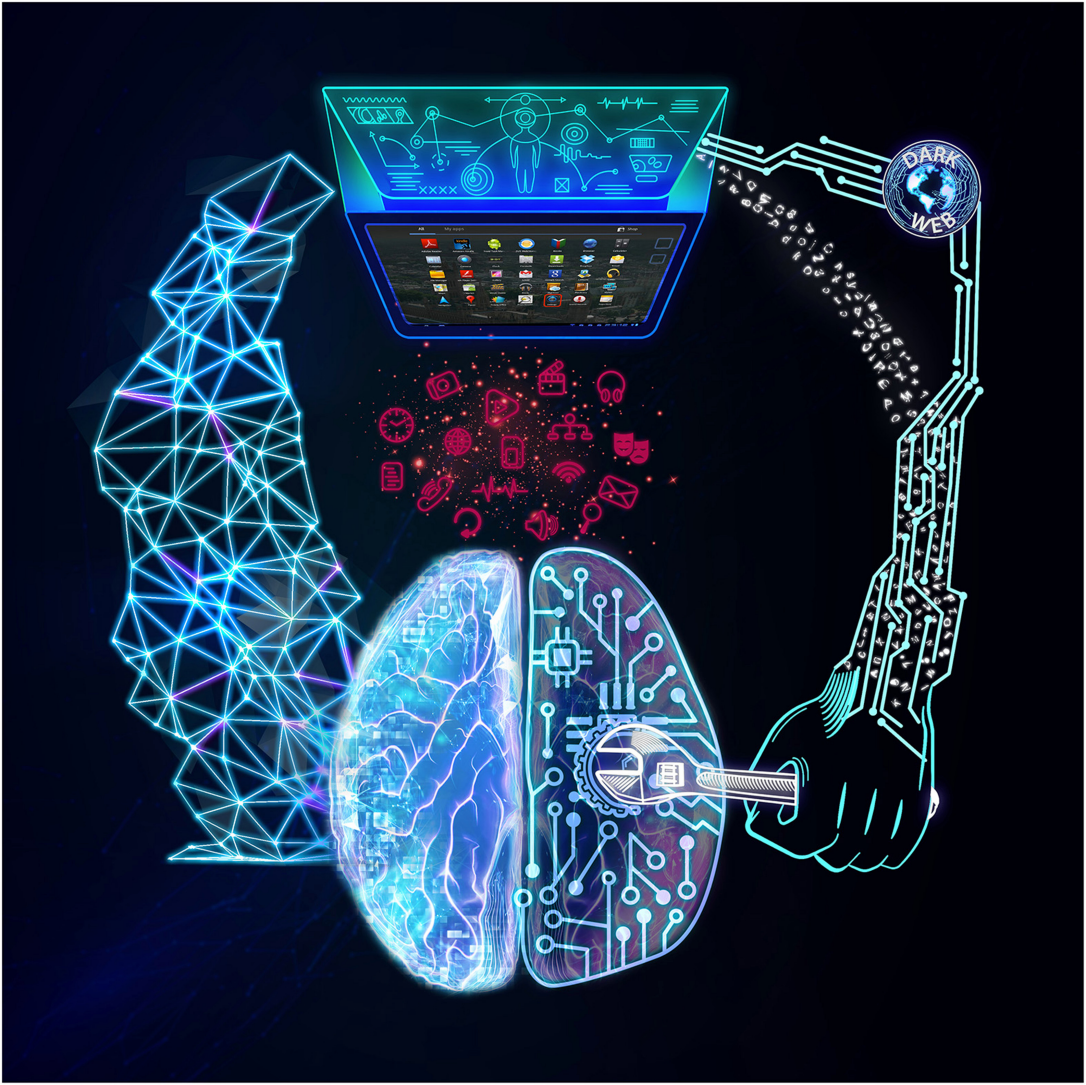
Digital Surveillance Capitalism. On the left, you see the potentially beneficial digital phenotyping, where clients' or patients' information are collected through their apps to better serve them. On the right, you see the potentially unintended consequences of the intersection of digital phenotyping, dark web, and digital neuromarketing where profiteers may use the collected personal information for their illegal and unethical financial gain. The figure also shows the bidirectional nature of these communications; such that, the dark web that is fed by digital phenotyping and digital neuromarketing; meanwhile, can simultaneously strengthen their hazardous impact.

The intersection of digital phenotyping and digital neuromarketing can be perilous and could potentially lead to what we may call “*digital surveillance capitalism”* in Figure 1. The former recognizes human preferences from the “inside” via multiple bodily sensors that may be out of our control, such as our heartbeat and other autonomic responses. The latter analyzes the input and directs us toward shopping for particular goods, voting for a particular candidate, or may steer us to a specific brand name. This kind of behavioral targeting also influences our concepts of free will, democracy, and our human agency in the long term. Moreover, targeting those with sub-optimal ability to control their impulses and interests, such as children and adolescents, could make this issue even more complicated. It can become even more complicated with integrating personal health information from the *dark web* as an illegal but potent data source (36). The dark web is a part of the interconnected network (internet) that requires specific softwares and configurations to access, where many illicit transactions of drugs, human organs, weapons, and data trade occur (37). For example, in 2016, health-related documents, including the family history of about 16 million individuals, were sold in the internet's black market (dark web) around 20-50$ (38). By Looking to this illegal data sharing and multiple similar uses of the dark web (39, 40), the undesireable kind of connection between digital neuromarketing and digital phenotyping would be strengthened (Figure 1).

To further clarify this menacing intersection, imagine a giant technology company or government agency accessing the individuals' digital phenotyping and neuromarketing data. This could potentially enable them to influence and manipulate a populations' emotions and collective external behavior. For example, prior to an election, they could present a seemingly casual political advertisement on their smartphone (digital neuromarketing). The population's reaction to these advertisements would subsequently be analyzed using digital biomarkers, such as a heartbeat change as a sign of satisfaction or disgust and anger (digital phenotyping). The next step would be to reinforce positive emotions toward the desired candidate and promote negative feelings toward the undesired candidate by targeting the individuals at the time of their relaxation and happiness with positive and relaxing ads supporting the desired candidate. Vise versa, during stressful moments, presenting them with negative advertisements against the undesired political candidate. Such scenarios would favor the emergence of powerful totalitarian governments that could control and shape the populations' external behavior based on their intrinsic reactions to targeted stimuli. A similar scenario could be extended to a big pharmaceutical company directing physicians and patients into a desired pharmaceutical choice, which would go on in many other contexts.

We believe that while digital phenotyping and digital neuromarketing will unravel many unknown frontiers of neuroscience and mental health, their intersection would act as a double-edged sword and bring serious mental health concerns to individuals, such as invasion of privacy, decrease in self-confidence, and use of unhealthy monitoring through digital phenotyping into mental health care programs. The following recommendations may be needed to be put in place to help reduce the unintended consequences.

- *Technical and Public Evaluation of technologies and media before release:* These advanced technologies should be rigorously studied and critically evaluated before their widespread implementation to maximize their benefits and minimize the potential misuse in the hands of profiteers. More rigorous, well-defined regulations and guidelines are urgently needed to protect the public from those who wish to exploit them for their financial or political gain. As an example, a single company has been penalized several times for privacy issues, the current regulations and laws seem unworkable (41–43). Besides, there are several technical methods to maintain the patients' and users' privacy. In parallel with using blockchain technology (44, 45), differential privacy algorithms are the most efficient methods currently utilized in this regard by creating random noise to sensitive data. In addition, other methods are also important; for instance, blurring face of bystanders and real-time processing locally to prevent the harms of data sharing with third parties (46). Also, “white hat hackers,” ethical professional hackers who use their knowledge and competency to find digital systems' vulnerability for good purposes (47), could play a significant role in evaluating apps that claim they do not collect and use this kind of information.
- *Regulatory processes in place with careful monitoring:* Independent mentoring and regulatory organizations should vigorously investigate abuses of these high technology tools, particularly digital phenotyping and neuromarketing, to protect the public from unethical practices. As suggested by multiple recent United Nations' documents and meetings (48, 49), establishing international collaborations and ethical committees to supervise and monitor digital tools' activity would be beneficial (44).
- *Transparency as a policy measure:* Each digital tool's ‘terms and policy' should clearly state that the applications may analyze the users' data and behavior for commercial intents as a separate item to be approved. It should be considered a crime if a company uses the consumers' data without their permission or without clearly explaining it to them, as this is a clear violation of consumers' right to privacy.
- *Public awareness and Education on apps:* The public should be educated on the benefits and the harms of using telehealth apps. Public health programs, NGOs, and human rights activists should take action on this. This is of more importance for those living in developing countries as they are at higher risk of being exploited in such unregulated intersections. Education should be contextual and need-based; thus, digital mental health and its related ethical considerations should be incorporated into the curriculum of mental health trainings to start this discussion early on with learners at all levels.
- *Taxing the information gathering:* Information is one of the most invaluable assets. Similar to property tax, we suggest information tax for giant companies to modulate the current inappropriate global trend toward compiling big data of the consumers. The notion of imposing such taxes will certainly draw the attention of politicians and policymakers to this important issue. We suggest such taxes be used for public education toward sustainable and ethical e-health solutions towards strengthening the health care system in low-resource settings.

## Data Availability Statement

The original contributions presented in the study are included in the article/supplementary material, further inquiries can be directed to the corresponding author/s.

## Author Contributions

HA conceived the concept and wrote the initial draft. All authors contributed to the evolving, editing, and revising of this paper and approved the final draft. All authors contributed to the article and approved the submitted version.

## Conflict of Interest

The authors declare that the research was conducted in the absence of any commercial or financial relationships that could be construed as a potential conflict of interest.

## Publisher's Note

All claims expressed in this article are solely those of the authors and do not necessarily represent those of their affiliated organizations, or those of the publisher, the editors and the reviewers. Any product that may be evaluated in this article, or claim that may be made by its manufacturer, is not guaranteed or endorsed by the publisher.
